# *GH1* Gene Polymorphisms Reveal Population-Level Allele Variation in North African (*Clarias gariepinus*) and Bighead Catfish (*Clarias macrocephalus*)

**DOI:** 10.3390/genes16111266

**Published:** 2025-10-27

**Authors:** Phonemany Thammachak, Piangjai Chalermwong, Chananya Patta, Wattanawan Jaito, Worapong Singchat, Thitipong Panthum, Trifan Budi, Kednapat Sriphairoj, Sittichai Hatachote, Prapansak Srisapoome, Narongrit Muangmai, Orathai Sawatdichaikul, Darren K. Griffin, Agostinho Antunes, Prateep Duengkae, Uthairat Na-Nakorn, Yoichi Matsuda, Kornsorn Srikulnath

**Affiliations:** 1 Animal Genomics and Bioresource Research Unit (AGB Research Unit), Faculty of Science, Kasetsart University, Bangkok 10900, Thailand; phonemamy.t@ku.th (P.T.); chananya.patt@ku.th (C.P.); wattanawan.ja@ku.th (W.J.); thitipong.pant@ku.ac.th (T.P.); trifan.bu@ku.th (T.B.); ffispssp@ku.ac.th (P.S.); ffisnrm@ku.ac.th (N.M.); d.k.griffin@kent.ac.uk (D.K.G.); prateep.du@ku.ac.th (P.D.); yoimatsu@agr.nagoya-u.ac.jp (Y.M.); 2 Department of Genetics, Faculty of Science, Kasetsart University, Chatuchak, Bangkok 10900, Thailand; 3 Special Research Unit for Wildlife Genomics (SRUWG), Department of Forest Biology, Faculty of Forestry, Kasetsart University, 50 Ngamwongwan, Chatuchak, Bangkok 10900, Thailand; 4 Faculty of Natural Resources and Agro-Industry, Kasetsart University Chalermphrakiat Sakon Nakhon Province Campus, Sakon Nakhon 47000, Thailand; kednapat.sr@ku.th (K.S.); sittichai.h@ku.th (S.H.); 5 Department of Aquaculture, Faculty of Fisheries, Kasetsart University, Bangkok 10900, Thailand; ffisurn@ku.ac.th; 6 Department of Fishery Biology, Faculty of Fisheries, Kasetsart University, Bangkok 10900, Thailand; 7 Department of Nutrition and Health, Institute of Food Research and Product Development, Kasetsart University, Bangkok 10900, Thailand; orathai.saw@ku.th; 8 School of Biosciences, University of Kent, Canterbury, Kent CT2 7, NJ, UK; 9 CIIMAR/CIMAR, Terminal de Cruzeiros do Portode Leixões, Interdisciplinary Centre of Marine and Environmental Research, University of Porto, 4450-208 Porto, Portugal; aantunes@ciimar.up.pt; 10 Department of Biology, Faculty of Sciences, University of Porto, 4169-007 Porto, Portugal; 11 Biodiversity Center, Kasetsart University (BDCKU), Bangkok 10900, Thailand

**Keywords:** allele, bighead catfish, *GH1* gene, North African catfish, purifying selection

## Abstract

Background/Objectives: North African catfish (*Clarias gariepinus*) and bighead catfish (*Clarias macrocephalus*) play crucial roles in Thai aquaculture. Although significant growth disparities exist among these species, the genetic factors underlying these differences are still unknown. This study aimed to identify *GH1* gene polymorphisms, in North African and bighead catfish populations across Thailand and Laos. Methods: Sequencing, phylogenetic, and clustering analyses were performed to assess genetic diversity, selection patterns, and lineage differentiation of catfish partial *GH1* fragment. Results: Six alleles of the studied fragment of *GH1* gene were identified; they differed at 33 variable sites within intron 2, located between the conserved regions at the 3′ end of exon 2 and the 5′ end of exon 3. At the population-level, *GH1* exhibited low heterozygosity (mean *H*_o_ = 0.043 ± 0.023; *H*_e_ = 0.059 ± 0.028). Bayesian clustering analyses identified two distinct genetic clusters, corresponding to North African and bighead catfish, apart from the bighead population in Laos, indicating their distinct genetic origins. Evidence of purifying selection was observed in both species. Phylogenetic analysis indicated the presence of lineage-specific alleles in the *GH1* gene. Conclusions: These findings provide valuable insights into *GH1* polymorphisms in commercially important catfish species and may help to develop future breeding programs aimed at enhancing aquaculture productivity.

## 1. Introduction

Fish are economically important as a source of animal protein and other valuable products. Consequently, owing to rising consumer demand and declining natural fish stocks, aquaculture has become a driving force in global food production. In Thailand, aquaculture is the cornerstone of the fisheries sector, contributing approximately 1 million tons annually to the national economy as of 2023 [1]. Among the contributors to this success in Thailand, a key component of domestic consumption is clariid catfish, which generates an annual value of THB 4353 billion (USD 128.87 billion) (15.41% of global aquaculture) being ranked in the top ten producers [1].

North African catfish (*C. gariepinus*) is originally native to various countries across the African continent. The fish, which has four pairs of thick-based barbels and an elongated head with a concave profile sloping downward from the snout to the nape, is described in the following manner. The head’s top is rough-textured, and three curved ridges, with the central one being the longest, mark the nape. The long, slender body has extended dorsal and anal fins. The upper body shows a marbled pattern of dark brownish-yellow, while the cheeks and belly are lighter. A pale vertical stripe is found at the caudal fin’s base, with fins darker and sometimes tinged with red at the edges. This species was introduced to Thailand in late 1987 [2] and is known for its strong adaptability and resistance to varying water conditions. These traits have led to this species widespread cultivation in Africa and other regions [3,4]. Thai consumers, however, do not favor North African catfish because of its unfavorable color and texture [4,5].

The bighead catfish (*C. macrocephalus*) is a freshwater species native to Thailand. The fish, characterized by an elongated, scaleless body and four pairs of barbels, exhibits coloration ranging from yellow to brown with black spots along the sides. It is described as yellowish and oily. The body shape is blunt, with a rounded occipital bone and a smooth skull. Sharp, rigid spines and long fin rays are present on the pectoral fins, while the underside from the chest to pelvic fins is white to yellow [4,5].

The hybrids of the mentioned two species currently dominate the market, accounting for a market share of over 90% in Thailand [6,7,8,9]. The technology was developed in 1987 to improve productivity and meat quality of catfish: hybrid catfish, produced via the artificial crossbreeding of male North African catfish and female bighead catfish (*C. macrocephalus*). Females of the hybrids, however, are partially sterile, whereas the hybrid males are completely sterile [4,10,11]. This necessitates the preparation of parental genetic stocks and resources for breeding. Nevertheless, the national production of hybrid catfish in Thailand has decreased from 124,463 tons in 2013 to 89,525 tons in 2023 [12]. The total production value is now estimated at approximately THB 3500–4000 million (USD 103–118 million), reflecting a 10.9% decline since 2022. The decline is attributed to issues such as low genetic stock quality, heat stress and climate effects, poor hatchery and water management, and high feed costs [13]. Additionally, the size of hybrid catfish gradually decreases with each generation, resulting in an extended growth period, from about 120 days to 150 days, to reach a marketable size [14,15,16,17]. Such declines in the survival, growth, conception, and fertility rates of North African and bighead catfish, as observed in their cultured populations, are attributed to uncontrolled management of breeding stock.

Growth performance is a crucial economic trait in aquaculture; it has the potential to enhance productivity and reduce costs. Accordingly, the difference in growth rates among North African, bighead, and hybrid catfish has been emphasized [4], and the difference has also been observed between ecotypes of North African catfish [18]. Various extrinsic factors, such as variations in feeding regimes and feeding durations, affect fish body weight gain by influencing the expression of muscle growth-related genes [19]. Thus, growth variations have a genetic basis owing to genetic polymorphisms associated with body size changes [19,20]. The growth in fish is regulated by a group of genes such as growth hormone (*GH*), growth hormone receptor (*GHR*), insulin-like growth factor (*IGF1*), myogenic regulatory factors (*MRFs*), and myostatin (*MSTN*) [19,21,22,23].

The *GH* gene is a key regulator of metamorphosis in fish and is associated with linear growth, feed utilization, and fasting metabolism, and plays a crucial role in the development of muscles and bones, as well as in other essential physiological processes [24]. GH, a 20.5 kDa polypeptide secreted by somatotrophs, is thus essential for growth and development in vertebrates and regulates cellular division, muscular growth, reproduction, osmoregulation, and food conversion in fish [24,25]. The *GH* gene is not structurally conserved among teleosts; carp (*Cyprinus carpio*) has five exons, whereas other teleosts, such as gilthead seabream (*Sparus aurata*), pacu (*Piaractus mesopotamicus*), and salmonine fishes, have six exons [26,27,28]. Additionally, the gene is duplicated as *GH1* and *GH2* in some fish such as salmonids, common carp, and tilapia [29,30]. Intraspecific polymorphisms in the *GH1* gene have been identified in several fish species, and these variations may affect growth and survival rates by altering the structure and function of GH [27,31,32]. Certain alleles of the *GH1* gene in tilapia and salmonids are associated with growth rate and adaptability to varying environmental conditions [33,34]. Among the clariid catfish species, the *GH1* gene of North African catfish (accession number: AF416488) contains five exons and four introns [35]. Furthermore, the amino acids of growth hormone tend to be conserved among clariid catfish species [36]. However, information pertaining to the correlation between *GH1* polymorphisms and growth performance in catfish is limited.

Given the significant difference in growth rate performance between North African and bighead catfish, we herein hypothesize that the alleles of the *GH1* gene may differ between the two species. Additionally, large-scale studies of genetic diversity using microsatellite genotyping have revealed high genetic variations in North African and bighead catfish in Thailand [9,37,38,39,40,41]. This suggests a high likelihood of *GH1* polymorphisms in catfish populations. Identification of polymorphisms of the *GH1* gene by complete or partial sequencing in various catfish species or ecotypes contributes to the development of effective tools, such as marker-assisted selection, for enhancing breeding programs. The findings can thus contribute to the advancement in catfish genetics and the development of effective selection strategies. Hence, this study aimed to identify interspecific or even intraspecific *GH1* gene polymorphisms.

## 2. Materials and Methods

### 2.1. Ethic Statement

The catfish included in this study were sourced from private farms or natural habitats. Caudal fin clip samples were obtained with prior authorization from fish farm owners or relevant governing authorities, and all individuals were immediately released back to their respective habitats after sampling. The Animal Experiment Committee at Kasetsart University reviewed and approved the animal use protocols for this study (Approval Nos. ACKU65-SCI-003, ACKU66-SCI-006, and ACKU66-SCI-014). The study was conducted in accordance with the ARRIVE guidelines (https://arriveguidelines.org) and complied with Kasetsart University’s Regulations on Animal Experiments.

### 2.2. Specimen Collection and DNA Extraction

A total of 589 captive and wild catfish specimens were collected from 16 locations in Thailand and Laos: research was conducted in three farmed North African catfish populations, four farmed bighead catfish populations, eight wild bighead catfish populations from Thailand, and one farmed bighead catfish population from Laos. Here we will briefly describe these samples, which included 3 North African (N = 256) and 13 bighead catfish populations (N = 333), and provide an additional information regarding sampling in Table S1. Tissue samples were collected from caudal fins (approximately 0.3 × 0.3 cm) of each individual, transferred into 1.5 mL tube containing 95% ethanol, and stored at 4 °C until use. Genomic DNA was isolated using the standard salting-out protocol, and its quality was assessed as described by Supikamolseni Et Al. [42].

### 2.3. PCR Amplification and Illumina^TM^ Short-Read Sequencing

Newly designed primers GH1_Catfish_F1 (5′-GAGCCTCGATAGAGTCCGA GT-3′) and GH1_Catfish_R1 (5′-TGTTACGACTTTGGCATTTCA-3′) were designed based on the sequences of North African catfish chromosome 28 (accession number: NC071127) targeting partial fragment of exon 2 to exon 3 of the *GH1* gene. SNPs within this region were found to be related to growth traits in fish [43]. The forward primer was modified by adding 8 bp sample-specific barcode tag sequences at its 5′-end (Macrogen Inc., Seoul, Korea). The PCR was performed using the Apsalagen PCR kit (Apsalagen Co., Ltd., Bangkok, Thailand) with 50 ng of genomic DNA under the following cycling conditions: initial denaturation at 95 °C for 10 min; 40 cycles of 95 °C for 30 s, 60 °C for 30 s, and 72 °C for 30 s; and a final extension at 72 °C for 5 min. Successful amplification was assessed by 1.5% agarose gel electrophoresis. A total of 589 samples were analyzed, which were processed in seven sequencing sets, each comprising 92 uniquely barcoded samples. These pooled libraries were sent for paired-end short-read (2 × 250 bp) sequencing on an Illumina NovaSeq™ 6000 platform at Novogene Co., Ltd. (Singapore). A single, clearly defined band corresponding to the expected amplicon size was observed, confirming specific amplification of the target *GH1* fragment. No nonspecific or additional bands, such as pseudogene or paralogous sequences, were detected.

### 2.4. Sequence Quality Control and Read Processing

Illumina paired-end reads were quality-assessed using FastQC v0.11.9 [44], retaining only reads with a Phred quality score (*q*) > 20. The AmpliSAS v1.0 pipeline [45] was employed for allele determination under the following parameters: minimum amplicon depth of 100, maximum of two alleles per individual, and degree of change criterion (DOC) based on sequencing depth. Remaining parameters followed default configurations as described by Lighten Et Al. [46]. Genotypes were classified as homozygous if a single dominant allele exceeded 80% frequency, or heterozygous if two distinct alleles were detected. Validated alleles were confirmed Via BLASTn (https://blast.ncbi.nlm.nih.gov/Blast.cgi) (accessed on 2 December 2024) tool in the Center for Biotechnology Information (NCBI) database. Sequences were aligned to the North African catfish *GH1* reference gene (accession number: AF416488) using Geneious Prime v2023.0.4 (https://www.geneious.com). The aligned sequences were then translated into amino acid sequences to detect any premature stop codons. Given that some *Clarias* species can exhibit segmental polyploidy or gene duplication [47], allelic assignment and filtering parameters in AmpliSAS were adjusted to minimize possible artifacts arising from paralogous sequences, ensuring that only true alleles of the *GH1* locus were retained.

### 2.5. Genetic Diversity and Data Analysis

Genetic diversity was evaluated by analyzing allelic frequency, number of alleles (*N*_a_), number of effective alleles (*N*_e_), observed heterozygosity (*H*_o_), expected heterozygosity (*H*_e_), and fixation index within populations (*F*) using GenAlEx version 6.5 [48] and allelic richness (*AR*) was calculated in FSTAT version 2.9.3 [49]. Nucleotide diversity (π) was calculated using DnaSP version 6.12 [50]. To further explore the genetic structure, *F*-statistics (*F*_IS_ and *F*_ST_) for the *GH1* gene were computed using FSTAT version 2.9.3 [51], as previously performed in Budi et al. [52,53]. Analysis of molecular variance (AMOVA) was carried out with Arlequin version 3.5.2.2 [54]. To visualize clusters of genetically related individuals, and discriminant analysis of principal components (DAPC) was performed in R version 4.3.2 [55] using the ADEGENET 2.0 package [56].

### 2.6. Phylogenetic Analysis

The *GH1* gene sequences obtained in this study were combined with Siluriform catfish sequences retrieved from NCBI using the BLASTn tool (https://blast.ncbi.nlm.nih.gov/Blast.cgi). BLASTn searches were performed using thresholds of >70% sequence identity and >85% query coverage. The optimal substitution model was determined with ModelFinder [57] based on the lowest Bayesian Information Criterion value. A maximum likelihood phylogenetic tree was then constructed using IQ-TREE version 1.6.12 [58] to visualize the evolutionary relationships among *GH1* gene alleles. Branch support was assessed Via ultrafast bootstrap analysis with 10,000 replicates, employing the Kimura 2-parameter (K2P) model for the *GH1* dataset. The phylogenetic tree topology was visualized using Interactive Tree of Life v5 [59].

### 2.7. Selection Analysis

The type of selection acting on the catfish *GH1* gene was assessed using the ratio of non-synonymous to synonymous substitution rates (*d*_N_/*d*_S_, *ω*). The *d*_N_/*d*_S_ was calculated using the Nei–Gojobori method [60] with the Jukes–Cantor correction, implemented in Molecular Evolutionary Genetics Analysis version 11 (MEGA 11) [61]. An *ω* value close to 1 indicates neutral selection, a value greater than 1 suggests positive selection, and a value less than 1 reflects purifying selection. In addition, neutrality tests including Tajima’s *D*, Fu and Li’s F*, and Fu and Li’s D* were performed using DnaSP version 6.12 [50] to further characterize the selection mode. To investigate potential selective sweeps, expected heterozygosity (*H*_e_) and the inbreeding coefficient (*F*_IS_) for the *GH1* gene were plotted based on genotyping data; a high *F*_IS_ combined with low *H*_e_ suggests a selective sweep or purifying selection, while a low *F*_IS_ with high *H*_e_ indicates neutral or balanced selection [62].

### 2.8. Multiple Sequence Alignment of GH1 Amino Acid Residues

Reference sequences of the *GH1* genes of different catfish species, Lanzhou catfish (*Silurus lanzhouensis*) (accession no. KM215221), blind catfish (*Rhamdia quelen*) (EF101341), yellowhead catfish (*Tachysurus fulvidraco*) (KU323395), channel catfish (*Ictalurus punctatus*) (AF267989 and S69215), iridescent shark catfish (*Pangasianodon hypophthalmus*) (JF303888 and HM137287), North African catfish (AF416488), Valencienne’s clariid (*Clarias dussumieri*) (HM485574), mudfish (*Clarias anguillaris*) (HM485573), and walking catfish (*Clarias batrachus*) (AF416486)], were retrieved from the NCBI using BLASTp (https://blast.ncbi.nlm.nih.gov/Blast.cgi). BLASTp analysis was performed in condition with thresholds of >60% sequence identity and >85% query coverage thresholds [63,64]. The amino acid residues of the *GH1* gene alleles identified in this study and reference sequences of other catfish species were aligned using ClustalW in Geneious Prime version 2023.0.4 (https://www.geneious.com). The alignment was then trimmed to 35 residues, and a maximum likelihood phylogenetic tree was constructed with 10,000 ultrafast bootstraps, as previously described.

## 3. Results

### 3.1. Genetic Diversity and Population Structure of Catfish Based on GH1 Gene

The 210 bp partial fragments of the *GH1* gene, ranging from the 3′ terminal region of exon 2 to the 5′ initial region of exon 3, were obtained from 589 individuals in three North African catfish and 13 bighead catfish populations (Table 1). The Cochrane-Armitage trend test (CATT) was employed, accounting for the relative frequencies within each category. The exact CATT has been demonstrated to be suitable for unbalanced sample sizes in 2 × C contingency tables. Thirty-three variable sites defining five newly identified alleles were observed in comparison to North African catfish *GH1* reference sequence (accession number: AF416488) (Table S2). One allele (CFGH*06) was shared with previously reported North African catfish sequences (accession numbers AF416488 and JAQMYH010000021). Allele CFGH*01 was the most common allele in North African and bighead catfish populations. All variable sites were positioned within intron 2; therefore, these changes did not affect the amino acid composition of the *GH1* gene. The mean *N*_a_ value per population was 1.625 ± 0.221, and the mean *π* value was 0.044 (Table 1). Positive *F*-values were observed for NYK-CG-C, KSN1-CG-C, KSN2-CG-C, SNK1-CM-W, and SNK2-CM-W, whereas SNK3-CM-W and LAO-CM-C exhibited negative *F* values. The mean *H*_o_ and *H*_e_ values were 0.043 ± 0.023 and 0.059 ± 0.028, respectively. Among North African catfish populations examined, the highest *H*_e_ value (0.378) was expected and highest *H*_o_ value (0.273) was observed in KSN1-CG, whereas NYK-CG-C showed the lowest *H*_e_ and *H*_o_ values (0.179 and 0.129, respectively). In bighead catfish populations, the highest *H*_e_ value was observed in SNK2-CM-W, and the lowest value was observed in SNK3-CM-W. The overall mean pairwise comparison of *H*_o_ and *H*_e_ values was statistically significant (*p* < 0.05). However, a pairwise comparison of the *H*_o_ and *H*_e_ in each population could not be performed because of the absence of diversity (*H*_o_ = 0) in several populations and the use of a single locus in this study (Tables S3–S5). All populations conformed to Hardy–Weinberg equilibrium, excepted (KSN1-CG-C, SNK2-CM-W, SNK1-CM-W) were show significant ** (*p* < 0.01) and *** (*p* < 0.001), respectively. The mean *AR* value was 1.625 ± 0.221, and the mean *N*_ea_ value was 1.082 ± 0.043. The mean *F*_IS_ value was 0.271 ± 0.178, and the *F*_ST_ values ranged from −0.110 to 0.899 (Table S6). The pairwise *F*_ST_ values between populations were not significantly different. Interpretation of population genetic parameters (*F*, *H*_e_, *H*_o_, *F*_IS_, *π*, *HWE*) requires caution for populations with small sample sizes—SPB1-CM-W (N = 5), SPB2-CM-W (N = 3), NPT1-CM-W (N = 2), NPT2-CM-W (N = 2), NST1-CM-W (N = 3), and NST2-CM-C (N = 10) or limited genetic variability, such as SNK4-CM-C (N = 25), STN-CM-C (N = 25), and NST2-CM-C (N = 10) (Table 1).

**Figure 1:**
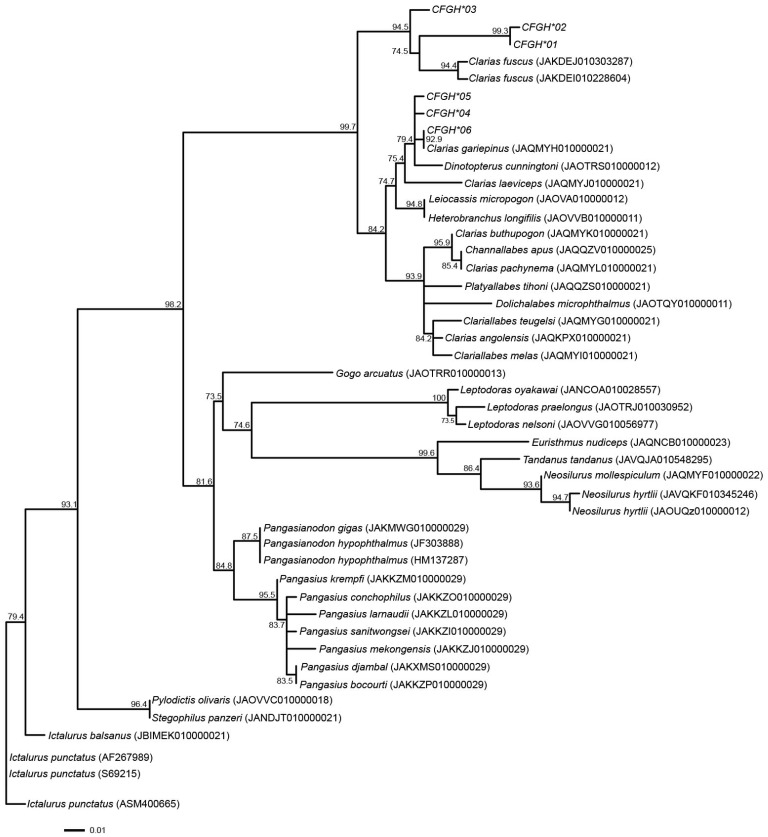
Maximum likelihood phylogenetic tree based on *GH1* gene alleles in catfish species. Values above branches represent bootstrap values.

Phylogenetic analysis based on maximum likelihood revealed well-supported clustering of *GH1* alleles within the *Clarias* genus. The alleles derived from *C. gariepinus* and *C. macrocephalus* grouped with other *Clarias* species such as *C. batrachus* and C*. dussumieri*, distinct from outgroup taxa of other genera (e.g., *Pangasianodon*, *Ictalurus*, *Silurus*). This pattern indicates that *GH1* alleles are genus-specific and supports the separation of the *Clarias* lineage from other Siluriformes. The tree topology (Figure 1) therefore reflects expected phylogenetic relationships based on previous nuclear and mitochondrial gene studies.

### 3.2. Selection and Selective-Sweep Analyses of Catfish Species

**Figure 2:**
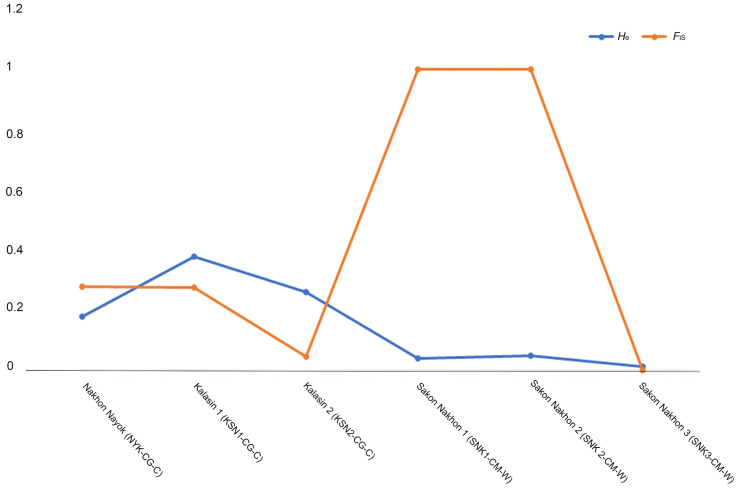
Mapping of expected heterozygosity (*H*_e_) against inbreeding coefficients (*F*_IS_) based on *GH1* gene for catfish populations.

Evidence of predominantly purifying selection was found in the *GH1* gene in North African and bighead catfish populations. Selective-sweep analysis revealed balanced selection, in which relatively high *H*_e_ over *F*_IS_ values in the KSN1-CG-C, KSN2-CG-C, and SNK3-CM-W populations are reflected. By contrast, the NYK-CG-C, SNK1-CM-W, and SNK2-CM-W populations exhibited higher *F*_IS_ values than *H*_e_, indicating that there were potential selective sweeps or purifying selection (Figure 2). However, a selective-sweep analysis could not be performed in most populations because *F*_IS_ values were not determined in several populations. Neutrality tests of *GH1* alleles revealed variations in Tajima’s *D*, Fu and Li’s D*, and Fu and Li’s F* values in North African and bighead catfish populations. Tajima’s *D* values ranged from −2.427 to 1.392 and were significant for the KSN1-CG-C, SNK1-CM-W, and SNK2-CM-W populations, whereas the values were not significant for the NYK-CG-C, KSN2-CG-C, SNK3-CM-W, SPB1-CM-W, and LAO-CM-C populations. The Fu and Li’s D* values ranging from −5.751 to 0.927 were insignificant for all populations except KSN1-CG-C and SNK1-CM-W. The Fu and Li’s F* values ranged from −5.170 to 1.038 and were insignificant for all populations except for KSN1-CG-C and SNK1-CM-W (Table 2). The *GH1* gene had an average *ω* value of 1.024, ranging from 0.300 to 1.189), indicating the presence of weak purifying selection in Kalasin 1 (KSN1-CG-C) population. The minimum *ω* value (0.3) was observed in Laos (LAO-CM-C) population. The maximum *ω* value (0.189) was observed in Kalasin 1 (KSN1-CG-C) population. However, in some populations the value could not be calculated for *GH1* sequences of North African and bighead catfish in Thailand and Laos, as no synonymous or nonsynonymous substitutions were observed in the regions of exons 2 and 3 examined (Table 3).

### 3.3. Multiple Sequence Alignment of GH1 Amino Acid Residues and Prediction

The sequence identities of 35 amino acids of the *GH1* gene with those of six alleles found in North African and bighead catfish populations examined in this study were 97.1% for blind catfish (*R*. *quelen*) (EF101341), yellowhead catfish (*T*. *fulvidraco*) (KU323395), and 100% for other seven catfish species, Lanzhou catfish (*S*. *lanzhouensis*) (accession no. KM215221), channel catfish (*I*. *punctatus*) (AF267989 and S69215), iridescent shark catfish (*P*. *hypophthalmus*) (JF303888 and HM137287), North African catfish (AF416488), Valencienne’s clariid (*Clarias dussumieri*) (HM485574), mudfish (*Clarias anguillaris*) (HM485573), and walking catfish (*Clarias batrachus*) (AF416486)] (Figure S2). This result indicates that the 35 amino acids are highly conserved in bighead catfish, North African catfish, and other catfish species. Similarly, maximum likelihood phylogenetic analysis based on amino acid residues revealed a monophyletic pattern of *GH1* amino acid residues within seven catfish species (Figures S3 and S4).

## 4. Discussion

Forty-two alleles were identified in the exon 2-intron-exon 3 region of *GH1* in 38 catfish species within Siluriform family, based on the number of species that could be matched using the BLASTn tool with the nucleotide sequences. Five new alleles that have not been observed in other fish species were identified in North African and bighead catfish population examined in this study. The *GH1* genes in both North African and bighead catfish populations exhibited low genetic diversity, and the diversity was lower in the bighead catfish populations. This suggests that the long-term adaptability and persistence of the *GH1* alleles should be considered in these catfish populations [65,66]. Potential selective sweeps were observed in the Nakhon Nayok population of North African catfish and in the Sakon Nakhon 1 and 2 populations of bighead catfish, which exhibited relatively higher *F*_IS_ values than *H*_e_ values. Selective sweeps explain how neutral polymorphisms are pulled near advantageous mutations, leading to reduced genetic diversity [67]. Our previous study using microsatellite genotyping, however, did not reveal any evidence of selective sweeps in these populations [40]. Therefore, selection pressure in the *GH1* gene may have affected allelic diversity in these three populations, resulting in an increase in their suitability to the environment. This is consistent with the highly conserved sequences found in partial exons 2 and 3 of *GH1* in the present study. Similar results could be seen in the NYK-CG-C population, which exhibited only one allele. The small sample sizes of some catfish populations may have been biased in estimating genetic diversity and understanding selective sweeps, warranting cautious interpretation. Nevertheless, the sample sizes used in this study fall within the minimally acceptable range for assessing genetic diversity and population structure in animal studies [68,69]. A framework is provided indicating that the sampling effort is sufficient to yield reliable insights into genetic diversity and population structure. Given the logistical and ethical considerations, fish populations are typically clustered based on small nuclear genome fragments, with population genomics or phylogenetics methods used to describe the methodology or present case studies for population structure analysis [70]. Neutrality tests using Tajima’s *D* and Fu and Li’s D* showed mostly positive values, suggesting that the variations in catfish populations may not be solely attributable to selection [71].

Phylogenetic analysis indicated that the alleles identified in this study formed a clade with the alleles from different clariid catfish species, including Hong Kong catfish (*C. fuscus*) and Singa (*Dinotopterus cunningtoni*). This aligned with the species phylogenetic trees based on other nuclear gene markers, such as *RAG1* and *RAG2*, and mitochondrial genome sequences [72,73]. This suggests the presence of lineage-specific alleles of *GH1*, although this result may have obtained by chance. Clustering analyses indicated that North African and bighead catfish populations were separated into different clusters across all *K*-values, except for the bighead catfish population from Laos. AMOVA revealed that the genetic variability of the *GH1* gene between populations was greater than that within populations. This strongly suggests that the two clusters have distinct genetic origins based on the partial sequence of *GH1*. The bighead population from Laos, however, exhibited a gene pool pattern similar to that of North African catfish populations, in contrast to the bighead population examined in Thailand. The existence of gene pools shared between North African and bighead catfish indicate that breeding and artificial selection during domestication or natural selection may not have fully driven the divergence of gene pools. Alternatively, genetic admixture or introgression of North African catfish might have occurred in the bighead population of Laos, although DNA barcoding and morphological evidence are likely to indicate otherwise. The samples of the Laos population were sourced from the market, raising the possibility of misidentification or contamination of hybrid catfish, because of uncertainty in morphological classification of hybrids [74]. Further analysis, using additional samples collected from different populations in various countries, is required to confirm the selection mode and correlation of North African and bighead catfish with growth traits. This will also help determine whether the distinctive gene pool is lineage-specific or a result of chance.

*GH1* expression is regulated by the pituitary-expressed transcription factor *PIT1*, which binds to the *GH1* proximal promoter and several introns [75]. In cattle and fish, genetic variations in the *GH1* intronic region are associated with productive traits [33,76,77]. An SNP in the first intron of the Nile tilapia *GH1* gene is considered to be related to growth rate [33]. In humans, the *GH1* gene consists of five exons and four introns, with relatively weak splice sites across introns 2 and 3, which potentially produce small amounts of smaller GH isoforms [78]. It remains unclear whether this intronic SNPs play a functional role in *GH1* expression or whether they undergo linkage disequilibrium with another functional SNPs. The SNP clusters associated with high growth rates is based on the concept of haplotype blocks defined as functional SNP sites grouped into blocks [78]. Such haplotype blocks of functional SNPs are considered to have been derived from a common ancestor. These structures can be carefully organized into distinct blocks of haplotype diversity. A small fraction of SNPs in each block, known as tag SNPs, represents a significant portion of haplotypes, thereby the screening of these fractions reducing the need to genotype all SNPs in association studies and fine-scale mapping of complex traits [79]. The present populations of North African and bighead catfish examined in this study have been formed through their own genetic drift and artificial and natural selections during the process of domestication of captive individuals, resulting in their shared yet distinct haplotype blocks and gene pool patterns. Identifying such lineage-specific haplotypes is hence essential for characterizing and maintaining chromosomal region-specific gene pools. This haplotype analysis may also be used for estimating the variance for quantitative traits among closely related individuals in genome-wide association studies [79,80,81]. Genetic variations in the *GH1* gene have been reported to be highly correlated with growth performance in fish species such as Nile tilapia, common carp, and Chinese lake gudgeons [32,76,82,83,84,85]. Gene interaction analyses in the current study, however, revealed no direct evidence that growth traits are linked to the intronic region of the *GH1* gene in North African and bighead catfish populations [27].

Genetic improvements have been achieved in classical breeding by using phenotypic culling and pedigree information. Molecular-assisted breeding enhances the efficiency of improvement by QTL mapping or marker-assisted selection with polymorphic DNA markers, leading to the expedition of outcomes and enabling the precise and early identification of superior phenotypes [86]. Sequencing of full length *GH1* gene containing five exons and four introns (approximately 1400 bp) poses challenges for short-read sequencing because short DNA fragments may underestimate genetic variation [87]. Sequence analysis using long-read sequencing technologies, such as Oxford Nanopore or PacBio, in larger cohort of catfish populations may validate these findings and enhance our understanding of *GH1* gene polymorphisms and their effects on growth traits.

## 5. Conclusions

Polymorphisms of the *GH1* genes of different catfish populations that are potentially related to growth performance were identified in this study. Six polymorphic alleles of the *GH1* gene, which are potentially related to growth performance, were identified in North African and bighead catfish populations examined in this study. The *GH1* gene, however, exhibited low diversity in the populations of both species. Two distinct genetic clusters, corresponding to North African and bighead catfish lineages, were identified, suggesting independent evolutionary origins of the species, except for the bighead catfish population in Laos. Furthermore, evidence of purifying selection was observed in some populations of both species. The finding of *GH1* polymorphism in North African and bighead catfish populations in Thailand serves as an initial step in understanding the genetic basis of growth rates between the species. It facilitates the identification of selective markers for breeding programs and effective genetic management of breeding stock. The highly conserved exonic regions and highly variable intronic regions observed in both species, nonetheless, pose challenges. Therefore, further study focusing on the association of growth traits with the polymorphic intronic regions of the *GH1* gene is required in North African and bighead catfish populations. Understanding the genetic basis of the *GH1* gene on growth rates in catfish provides valuable insights for developing molecular breeding methods to improve the growth rate of the locally favored bighead catfish.

## Figures and Tables

**Table 1:** Nucleotide sequence diversity in catfish populations in Thailand and Laos based on *GH1* sequences.

Species	Population	Code	*N*	*N*_a_	*N*_e_	*AR*	*H*_o_	*H*_e_	*F*	*F*_IS_	*π*	HWE
*C. gariepinus*	Nakhon Nayok	NYK-CG-C	31	3.000	1.218	3.000	0.129	0.179	0.279	0.279	0.0027 ± 0.0008	0.79 ^ns^
	Kalasin 1	KSN1-CG-C	88	4.000	1.607	4.000	0.273	0.378	0.278	0.277	0.0053 ± 0.0015	0.003 **
	Kalasin 2	KSN2-CG-C	137	2.000	1.351	2.000	0.248	0.260	0.044	0.046	0.0031 ± 0.0003	0.607 ^ns^
*C. macrocephalus*	Sing Buri	SBR-CM-C	19	1.000	1.000	1.000	0.000	0.000	-	-	0	-
	Sakon Nakhon 1	SNK1-CM-W	49	2.000	1.042	2.000	0.000	0.040	1.000	1.000	0.0022 ± 0.0021	0.000 ***
	Sakon Nakhon 2	SNK2-CM-W	78	2.000	1.053	2.000	0.000	0.050	1.000	1.000	0.0026 ± 0.0017	0.000 ***
	Sakon Nakhon 3	SNK3-CM-W	82	2.000	1.012	2.000	0.012	0.012	−0.006	0.000	0.0001 ± 0.0001	0.956 ^ns^
	Sakon Nakhon 4	SNK4-CM-C	25	1.000	1.000	1.000	0.000	0.000	-	-	0	-
	Suphan Buri 1	SPB1-CM-W	5	1.000	1.000	1.000	0.000	0.000	-	-	0	-
	Suphan Buri 2	SPB2-CM-W	3	1.000	1.000	1.000	0.000	0.000	-	-	-	-
	Nakhon Pathom 1	NPT1-CM-W	2	1.000	1.000	1.000	0.000	0.000	-	-	-	-
	Nakhon Pathom 2	NPT2-CM-W	2	1.000	1.000	1.000	0.000	0.000	-	-	-	-
	Nakhon Si Thammarat 1	NST1-CM-W	3	1.000	1.000	1.000	0.000	0.000	-	-	-	-
	Nakhon Si Thammarat 2	NST2-CM-C	10	1.000	1.000	1.000	0.000	0.000	-	-	0	-
	Surat Thani	STN-CM-C	25	1.000	1.000	1.000	0.000	0.000	-	-	0	-
	Laos	LAO-CM-C	30	2.000	1.034	2.000	0.033	0.033	−0.017	-	0.0005 ± 0.0005	0.926 ^ns^
	Overall	-	589	1.625	1.082	1.625	0.043	0.059	0.368	0.271	0.04402	-
	SD	-	-	±0.221	±0.043	±0.221	±0.023	±0.028	±0.112	±0.178	-	-

*N*, number of individuals; *N*_a_, number of different alleles; *N*_e_, number of effective alleles; *H*_e_, expected heterozygosity; *H*_o_, observed heterozygosity; *AR*, allelic richness; *F*, fixation index; *F*is, inbreeding coefficient related to subpopulations; π, nucleotide diversity; HWE, Hardy–Weinberg equilibrium (HWE). ** (*p* < 0.01); *** (*p* < 0.001); ns, not significant.

**Table 2:** The results of neutrality test for the *GH1* gene sequences in the North African and bighead catfish populations in Thailand and the bighead catfish population in Laos.

Species	Population	Code	Tajima’s *D*	Fu and Li’s *D*	Fu and Li’s *F*
***C. gariepinus***	Nakhon Nayok	NYK-CG-C	−0.382 ^ns^	0.927 ^ns^	0.631 ^ns^
	Kalasin 1	KSN1-CG-C	−1.95 *	−5.751 **	−5.17 **
	Kalasin 2	KSN2-CG-C	1.392 ^ns^	0.647 ^ns^	1.038 ^ns^
***C. macrocephalus***	Sing Buri	SBR-CM-C	-	-	-
	Sakon Nakhon 1	SNK1-CM-W	−2.427 **	−4.974 **	−4.875 **
	Sakon Nakhon 2	SNK2-CM-W	−2.074 *	−1.473 ^ns^	0.275 ^ns^
	Sakon Nakhon 3	SNK3-CM-W	−1.044 ^ns^	−1.991 ^ns^	−1.988 ^ns^
	Sakon Nakhon 4	SNK4-CM-C	-	-	-
	Suphan Buri 1	SPB1-CM-W	−1.093 ^ns^	−1.093 ^ns^	−1.113 ^ns^
	Suphan Buri 2	SPB2-CM-W	-	-	-
	Nakhon Pathom 1	NPT1-CM-W	-	-	-
	Nakhon Pathom 2	NPT2-CM-W	-	-	-
	Nakhon Si Thammarat 1	NST1-CM-W	-	-	-
	Nakhon Si Thammarat 2	NST2-CM-C	-	-	-
	Surat Thani	STN-CM-C	-	-	-
	Laos	LAO-CM-C	−1.503 ^ns^	−2.319 ^ns^	−2.408 ^ns^
	*C. gariepinus*	-	−1.827 *	−7.310 **	−6.174 **
	*C. macrocephalus*	-	−0.312 ^ns^	0.972 ^ns^	0.523 ^ns^
	Overall mean value	-	−2.093 *	−8.383 **	−6.267 **

ns, not significant; *, *p* < 0.05; **, *p* < 0.01.

**Table 3:** Rates of synonymous (*d*_S_) and nonsynonymous (*d*_N_) substitutions in nucleotide sequences of *GH1* gene in Catfish populations.

Species	Population	Code	*N*	*d_S_ (±SE)*	*d_N_ (±SE)*	*ω (d_N_*/*d_S_)*	*Z-Test*	
							*p-Value*	*Z-Value*
***C. gariepinus***	Nakhon Nayok	NYK-CG-C	31	0.000 ± 0.000	0.013 ± 0.008	-	0.075	1.794
	Kalasin 1	KSN1-CG-C	88	0.337 ± 0.103	0.401 ± 0.078	1.189	0.061	1.891
	Kalasin 2	KSN2-CG-C	137	0.000 ± 0.000	0.014 ± 0.010	-	0.159	1.417
***C. macrocephalus***	Sing Buri	SBR-CM-C	19	-	-	-	-	
	Sakon Nakhon 1	SNK1-CM-W	49	0.376 ± 0.118	0.263 ± 0.666	0.699	0.991	0.011
	Sakon Nakhon 2	SNK2-CM-W	78	0.376 ± 0.118	0.263 ± 0.062	0.699	0.991	0.011
	Sakon Nakhon 3	SNK3-CM-W	82	0.000 ± 0.000	0.0070 ± 0.006	-	0.322	0.994
	Sakon Nakhon 4	SNK4-CM-C	25	-	-	-	-	-
	Suphan Buri 1	SPB1-CM-W	5	-	-	-	-	-
	Suphan Buri 2	SPB2-CM-W	3	-	-	-	-	-
	Nakhon Pathom 1	NPT1-CM-W	2	-	-	-	-	-
	Nakhon Pathom 2	NPT2-CM-W	2	-	-	-	-	-
	Nakhon Si Thammarat 1	NST1-CM-W	3	-	-	-	-	-
	Nakhon Si Thammarat 2	NST2-CM-C	10	-	-	-	-	-
	Surat Thani	STN-CM-C	25	-	-	-	-	-
	Laos	LAO-CM-C	30	0.020 ± 0.021	0.006 ± 0.006	0.3	0.583	−0.550
	Overall mean	-	589	0.485 ± 0.120	0.497 ± 0.093	1.024	0.071	1.822

Number of individuals (*N*).

## Data Availability

The full dataset and metadata in this study are available from the Dryad Digital Repository (https://doi.org/10.5061/dryad.sbcc2frhz) (registered in the database on 15 January 2025). All sequences were deposited in the National Center for Biotechnology Information (NCBI) (https://www.ncbi.nlm.nih.gov/; accession number: PQ877069–PQ877074 (deposited on 14 January 2025).

## Supplementary Materials

The following supporting information can be downloaded at https://www.mdpi.com/article/10.3390/genes16111266/s1: Figure S1: Discriminant analysis of principal components (DAPC) of three North African catfish and 12 bighead catfish populations in Thailand and one bighead catfish population in Laos. Each population is plotted by different color; Figure S2: Alignment of partial amino acid sequences of the *GH1* gene (35 amino acids), covering the 3′ terminal region of exon 2 and the 5′ initial region of exon 3, among six allelic sequences found for 16 catfish populations (*Clarias gariepinus and C. macrocephalus*) in Thailand and Laos and reference sequences of nine catfish species; Figure S3: GH1 protein secondary structure production in North African and bighead catfish species in Thailand and Laos; Figure S4: Maximum likelihood phylogenetic tree based on *GH1* gene amino acid residues in catfish species. The length of bars represents substitution per site value; Table S1: Details of the catfish specimens used in this study; Table S2: Variable sites in the sequences of six *GH1* alleles of North African and bighead catfish populations in Thailand and Laos; Table S3: Comparison of observed (*H*_o_) and expected heterozygosity (*H*_e_) of the *GH1* gene in catfish populations; Table S4: Comparison of expected heterozygosity (*H*_e_) of the *GH1* gene between catfish populations; Table S5: Comparison of observed heterozygosity (*H*_o_) of the *GH1* gene between catfish populations; Table S6: Genetic differentiation between catfish populations in Thailand and Laos based on the *GH1* sequences; Table S7: The results of Analysis of Molecular Variance (AMOVA) for 16 catfish populations in Thailand and Laos.
