# Supplementary material for: GH1 Gene Polymorphisms Reveal Population-Level Allele Variation in North African (Clarias gariepinus) and Bighead Catfish (Clarias macrocephalus)
Source: Genes (Basel). 2025 Oct 27;16(11):1266. doi: 10.3390/genes16111266 (PMC12652214; doi:10.3390/genes16111266)
Supplement: Supplementary file 1 [file genes-16-01266-s001.zip › genes-3923724-supplementary.pdf]

Supplementary Legends

Supplementary Figures

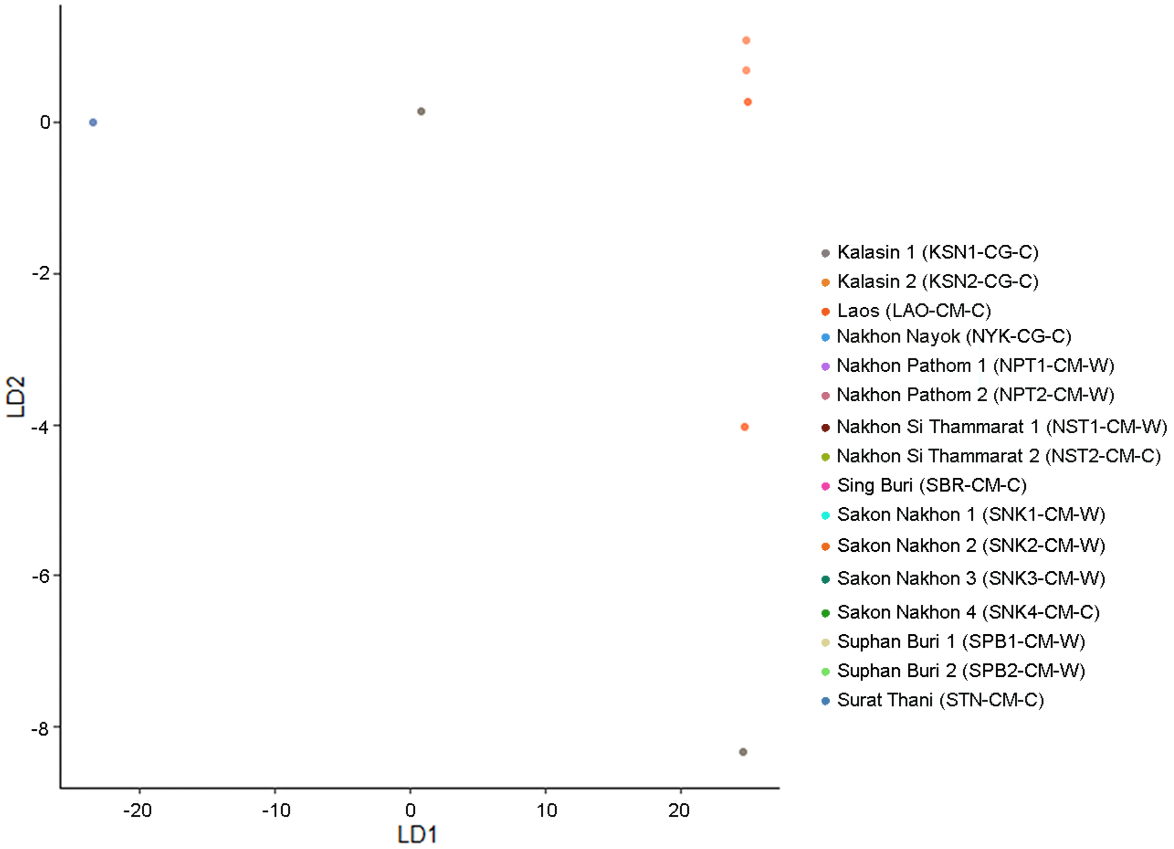

Fig S1 Discriminant analysis of principal components (DAPC) of three North African catfish and 12 bighead catfish populations in Thailand and one bighead catfish population in Laos. Each population is plotted by different color.

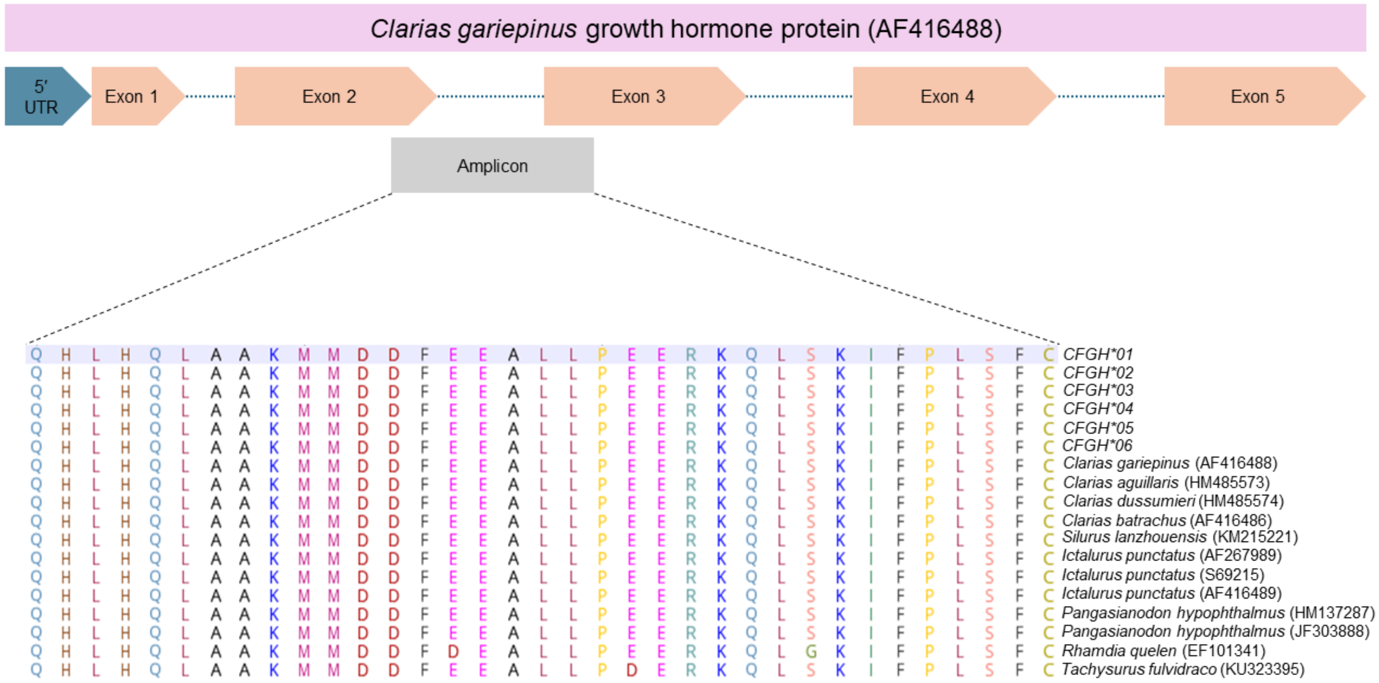

Fig S2 Alignment of partial amino acid sequences of the *GH1* gene (35 amino acids), covering the 3' terminal region of exon 2 and the 5' initial region of exon 3, among six allelic sequences found for 16 catfish populations (*Clarias gariepinus* and *C. macrocephalus*) in Thailand and Laos and reference sequences of nine catfish species.

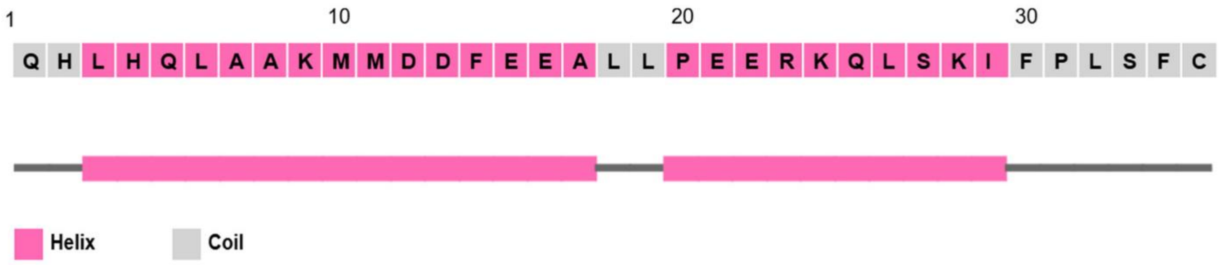

Fig S3 GH1 protein secondary structure production in North African and bighead catfish species in Thailand and Laos.

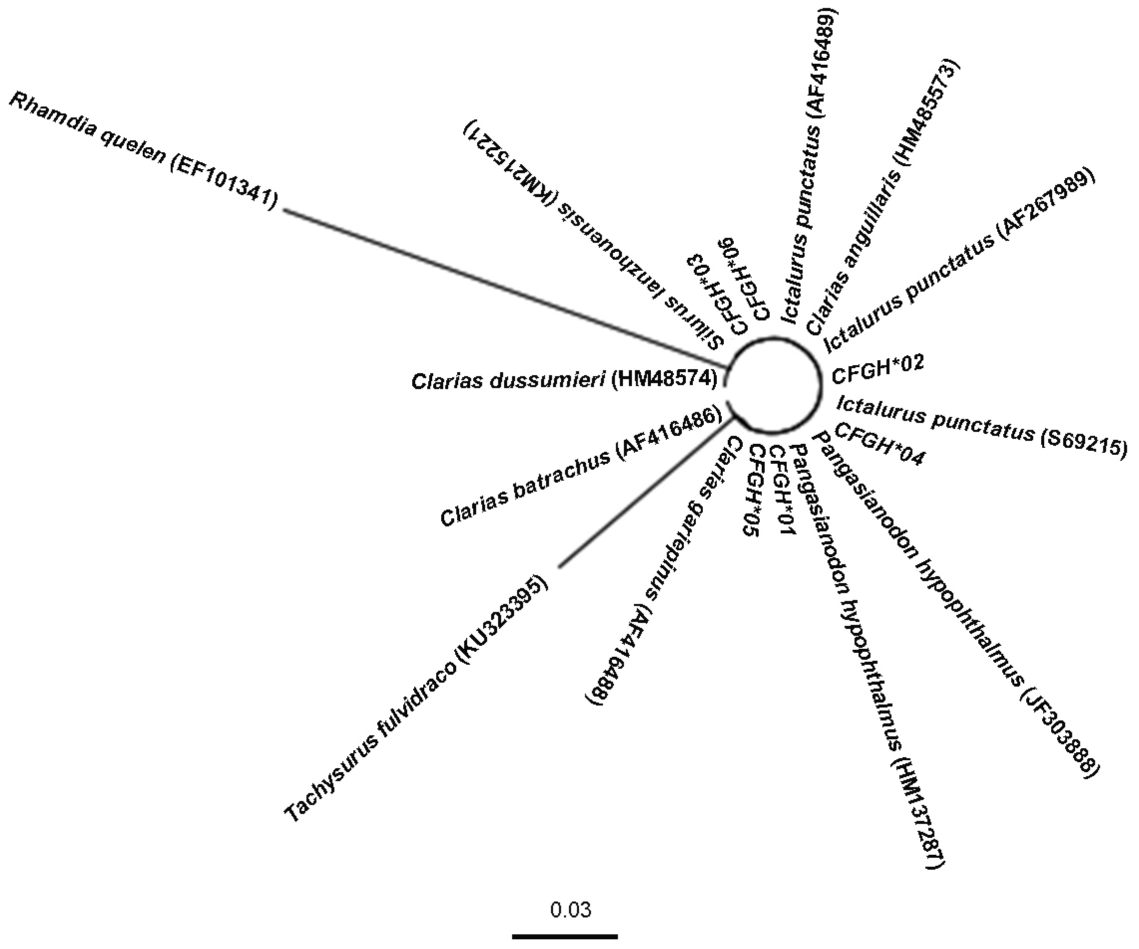

Fig S4 Maximum likelihood phylogenetic tree based on *GH1* gene amino acid residues in catfish species. The length of bars represents substitution per site value.

Supplementary Table

Table S1 Details of the catfish specimens used in this study

Species	Population	Code	*N**	Location	Subdistrict	District	Province	GPS coordinate
C. gariepinus	Nakhon Nayok	NYK-CG-C	31	Jeed Jad Farm	Si Chula	Mueang Nakhon Nayok	Nakhon Nayok	14˚05’58"N, 101˚09’39"E
	Kalasin 1	KSN1-CG-C	88	Kalasin Fish Farm (Betagro), Phu Sing	Buaban	Yangtalad Kalasin	Kalasin	16˚39’29"N, 103˚29’10"E
	Kalasin 2	KSN2-CG-C	137	Kalasin Fish Farm (Betagro), Phu Sing	Buaban	Yangtalad Kalasin	Kalasin	16˚39’29"N, 103˚29’10"E
C. macrocephalus	Sing Buri	SBR-CM-C	19	Phakin fish species	Thap Ya	In Buri	Sing Buri	14˚58’13"N, 100˚18’41"E
	Sakon Nakhon 1	SNK1-CM-W	49	Nong Han	That Choeng Chum	Mueang Sakon Nakhon	Sakon Nakhon	17°21’14"N, 104°19’16"E
	Sakon Nakhon 2	SNK2-CM-W	78	Susarn Donkajo	That Choeng Chum	Mueang Sakon Nakhon	Sakon Nakhon	17°14’05"N, 104°12’28"E
	Sakon Nakhon 3	SNK3-CM-W	82	Barnthavat	Laopordang	Mueang Sakon Nakhon	Sakon Nakhon	17°14’46"N, 104°23’82"E
	Sakon Nakhon 4	SNK4-CM-C	25	Fisheries Research and Development center Sakon Nakhon	That Choengchum	Mueang Sakon Nakhon	Sakon Nakhon	17°17’06"N, 104°15’24"E
	Suphan Buri 1	SPB1-CM-W	5	Khlong Ban Chang	Ban Chang	Song Phi Nong	Suphan Buri	14°14’57.1"N, 100°11’13.0"E
	Suphan Buri 2	SPB2-CM-W	3	Khlong Bang Khan Chang	Bang Ta then	Song Phi Nong	Suphan Buri	14°14’40.5"N, 100°07’60.0"E
	Nakhon Pathom 1	NPT1-CM-W	2	Khlong Mon Thong	Huai Mon Thong	Kamphaeng Saen	Nakhon Pathom	13°59’17.7"N, 99°56’37.3"E
	Nakhon Pathom 2	NPT2-CM-W	2	Tha Chin Rever Basin	Bang Len	Nakhon Chai Si	Nakhon Pathom	13°88’01"N, 100°24’17"E
	Nakhon Si Thammarat 1	NST1-CM-W	3	Pak Phanang River	Wang Ang	Cha-uat	Nakhon Si Thammarat	7°58’12"N, 99°58’12"E
	Nakhon Si Thammarat 2	NST2-CM-C	10	Nakhon Si Thammarat Pond	Phrommalok	Phrom Khiri	Nakhon Si Thammarat	8°61’27"N, 100°17’03"E
	Surat Thani	STN-CM-C	25	Fisheries Center Surat Thani	Tha Kham	Phunphin	Surat Thani	9°15’92"N, 99°42’84"E
	Laos	LAO-CM-C	30	Na Nga market	Na Nga	Naxaythong	Vientiane	18°02’90"N, 102°57’65"E

**N*, number of samples

Tables S2 Variable sites in the sequences of six *GH1* alleles of North African and bighead catfish populations in Thailand and Laos

**Nucleotide position in the *GH1* gene of *Clarias gariepinus* (AF416488)**		**Variable sites of alleles**					
		CFGH*01	***CFGH*02***	***CFGH*03***	***CFGH*04***	***CFGH*05***	***CFGH*06***
433	A	.	.	T	T	T	.
436	C	A	A	A	.	.	.
440	A	G	G	G	.	.	.
441	G	-	-	T	.	.	.
442	A	T	T	T	.	.	.
444	T	.	.	C	.	.	.
445	G	A	A	A	.	.	.
446	A	.	.	T	.	.	.
451	A	G	G	.	.	.	.
452	G	.	A	.	.	.	.
453	C	T	T	.	.	.	.
466	T	.	.	C	.	.	.
467	A	-	-	-	.	.	.
468	C	-	-	-	.	.	.
469	A	-	-	-	.	.	.
470	G	-	-	-	.	.	.
471	T	-	-	-	.	.	.
478	C	T	T	T	.	.	.
484	A	C	C	C	.	.	.
485	G	A	A	.	.	.	.
496	T	C	C	C	.	.	.
498	A	C	C	.	.	.	.
501	T	C	C	C	.	.	.
505	T	C	C	.	.	.	.
510	T	C	C	C	.	.	.
513	C	.	.	T	.	.	.
518	T	.	.	G	.	.	.
521	A	G	G	.	.	.	.
525	C	.	.	.	T	.	.
526	G	T	T	T	.	.	.
529	T	C	C	.	.	.	.
536	G	.	.	.	.	A	.
546	A	-	-	-	.	.	.
*N**	-	327	1	3	281	20	57

*N**, number of samples

Table S3 Comparison of observed (H_o) and expected heterozygosity (H_e) of the *GH1* gene in catfish populations

**Populations**	**Difference of mean values(?)**	**Standard error**	***t*-test**	***p*-value**
SNK3-CM-W	0	0	NaN	NaN
SBR-CM-C	0	0	NaN	NaN
KSN1-CG-C	−0.105	0	−Inf	NaN
NYK-CG-C	−0.05	0	−Inf	NaN
SNK4-CM-C	0	0	NaN	NaN
SNK2-CM-W	−0.05	0	−Inf	NaN
SNK1-CM-W	−0.04	0	−Inf	NaN
SPB1-CM-W	0	0	NaN	NaN
SPB2-CM-W	0	0	NaN	NaN
NPT1-CM-W	0	0	NaN	NaN
NPT2-CM-W	0	0	NaN	NaN
NST1-CM-W	0	0	NaN	NaN
NST2-CM-W	0	0	NaN	NaN
STN-CM-C	0	0	NaN	NaN
KSN2-CG-C	−0.012	0	−Inf	NaN
LAO-CM-C	0	0	NaN	NaN

Inf, infinitive; NaN, not a number

Table S4 Comparison of expected heterozygosity (H_e) of the *GH1* gene between catfish populations

Population 1	Population 2	Difference of mean	Standard error	*t*-test	*p*-value
SNK3-CM-W	SBR-CM-C	0.012	0	Inf	NaN
SNK3-CM-W	KSN1-CG-C	−0.366	0	−Inf	NaN
SNK3-CM-W	NYK-CG-C	−0.167	0	−nf	NaN
SNK3-CM-W	SNK4-CM-C	0.012	0	Inf	NaN
SNK3-CM-W	SNK2-CM-W	−0.038	0	−Inf	NaN
SNK3-CM-W	SNK1-CM-W	−0.028	0	−Inf	NaN
SNK3-CM-W	SPB1-CM-W	0.012	0	Inf	NaN
SNK3-CM-W	SPB2-CM-W	0.012	0	Inf	NaN
SNK3-CM-W	NPT1-CM-W	0.012	0	Inf	NaN
SNK3-CM-W	NPT2-CM-W	0.012	0	Inf	NaN
SNK3-CM-W	NST1-CM-W	0.012	0	Inf	NaN
SNK3-CM-W	NST2-CM-W	0.012	0	Inf	NaN
SNK3-CM-W	STN-CM-C	−0.248	0	Inf	NaN
SNK3-CM-W	KSN2-CG-C	−0.021	0	−Inf	NaN
SNK3-CM-W	LAO-CM-C	−0.096	0	−Inf	NaN
SBR-CM-C	KSN1-CG-C	−0.378	0	−Inf	NaN
SBR-CM-C	NYK-CG-C	−0.179	0	−Inf	NaN
SBR-CM-C	SNK4-CM-C	0	0	NaN	NaN
SBR-CM-C	SNK2-CM-W	−0.05	0	−Inf	NaN
SBR-CM-C	SNK1-CM-W	−0.04	0	−Inf	NaN
SBR-CM-C	SPB1-CM-W	0	0	NaN	NaN
SBR-CM-C	SPB2-CM-W	0	0	NaN	NaN
SBR-CM-C	NPT1-CM-W	0	0	NaN	NaN
SBR-CM-C	NPT2-CM-W	0	0	NaN	NaN
SBR-CM-C	NST1-CM-W	0	0	NaN	NaN
SBR-CM-C	NST2-CM-W	0	0	NaN	NaN
SBR-CM-C	STN-CM-C	0	0	NaN	NaN
SBR-CM-C	KSN2-CG-C	−0.26	0	−Inf	NaN
SBR-CM-C	LAO-CM-C	−0.033	0	−Inf	NaN
KSN1-CG-C	NYK-CG-C	0.199	0	Inf	NaN
KSN1-CG-C	SNK4-CM-C	0.378	0	Inf	NaN
KSN1-CG-C	SNK2-CM-W	0.328	0	Inf	NaN
KSN1-CG-C	SNK1-CM-W	0.338	0	Inf	NaN
KSN1-CG-C	SPB1-CM-W	0.378	0	Inf	NaN
KSN1-CG-C	SPB2-CM-W	0.378	0	Inf	NaN
KSN1-CG-C	NPT1-CM-W	0.378	0	Inf	NaN

**Population 1**	**Population 2**	**Difference of mean**	**Standard error**	***t*-test**	***p*-value**
KSN1-CG-C	NPT2-CM-W	0.378	0	Inf	NaN
KSN1-CG-C	NST1-CM-W	0.378	0	Inf	NaN
KSN1-CG-C	NST2-CM-W	0.378	0	−Inf	NaN
KSN1-CG-C	STN-CM-C	0.378	0	Inf	NaN
KSN1-CG-C	KSN2-CG-C	0.118	0	Inf	NaN
KSN1-CG-C	LAO-CM-C	0.345	0	Inf	NaN
NYK-CG-C	SNK4-CM-C	0.179	0	Inf	NaN
NYK-CG-C	SNK2-CM-W	0.129	0	Inf	NaN
NYK-CG-C	SNK1-CM-W	0.139	0	Inf	NaN
NYK-CG-C	SPB1-CM-W	0.179	0	Inf	NaN
NYK-CG-C	SPB2-CM-W	0.179	0	Inf	NaN
NYK-CG-C	NPT1-CM-W	0.179	0	Inf	NaN
NYK-CG-C	NPT2-CM-W	0.179	0	Inf	NaN
NYK-CG-C	NST1-CM-W	0.179	0	Inf	NaN
NYK-CG-C	NST2-CM-W	0.179	0	Inf	NaN
NYK-CG-C	STN-CM-C	0.179	0	Inf	NaN
NYK-CG-C	KSN2-CG-C	−0.081	0	−Inf	NaN
NYK-CG-C	LAO-CM-C	0.146	0	Inf	NaN
SNK4-CM-C	SNK2-CM-W	−0.05	0	−Inf	NaN
SNK4-CM-C	SNK1-CM-W	−0.04	0	−Inf	NaN
SNK4-CM-C	SPB1-CM-W	0	0	NaN	NaN
SNK4-CM-C	SPB2-CM-W	0	0	NaN	NaN
SNK4-CM-C	NPT1-CM-W	0	0	NaN	NaN
SNK4-CM-C	NPT2-CM-W	0	0	NaN	NaN
SNK4-CM-C	NST1-CM-W	0	0	NaN	NaN
SNK4-CM-C	NST2-CM-W	0	0	NaN	NaN
SNK4-CM-C	STN-CM-C	0	0	NaN	NaN
SNK4-CM-C	KSN2-CG-C	−0.26	0	−Inf	NaN
SNK4-CM-C	LAO-CM-C	−0.033	0	−Inf	NaN
SNK2-CM-W	SNK1-CM-W	0.01	0	Inf	NaN
SNK2-CM-W	SPB1-CM-W	0.05	0	Inf	NaN
SNK2-CM-W	SPB2-CM-W	0.05	0	Inf	NaN
SNK2-CM-W	NPT1-CM-W	0.05	0	Inf	NaN
SNK2-CM-W	NPT2-CM-W	0	0	Inf	NaN
SNK2-CM-W	NST1-CM-W	0.05	0	Inf	NaN
SNK2-CM-W	NST2-CM-W	0.05	0	Inf	NaN
SNK2-CM-W	STN-CM-C	0.05	0	Inf	NaN

Population 1	Population 2	Difference of mean	Standard error	*t*-test	*p*-value
SNK2-CM-W	KSN2-CG-C	−0.21	0	−Inf	NaN
SNK2-CM-W	LAO-CM-C	0.017	0	Inf	NaN
SNK1-CM-W	SPB1-CM-W	0.04	0	Inf	NaN
SNK1-CM-W	SPB2-CM-W	0.04	0	Inf	NaN
SNK1-CM-W	NPT1-CM-W	0.04	0	Inf	NaN
SNK1-CM-W	NPT2-CM-W	0.04	0	Inf	NaN
SNK1-CM-W	NST1-CM-W	0.04	0	Inf	NaN
SNK1-CM-W	NST2-CM-W	0.04	0	Inf	NaN
SNK1-CM-W	STN-CM-C	0.04	0	Inf	NaN
SNK1-CM-W	KSN2-CG-C	−0.22	0	−Inf	NaN
SNK1-CM-W	LAO-CM-C	0.007	0	Inf	NaN
SPB1-CM-W	SPB2-CM-W	0	0	NaN	NaN
SPB1-CM-W	NPT1-CM-W	0	0	NaN	NaN
SPB1-CM-W	NPT2-CM-W	0	0	NaN	NaN
SPB1-CM-W	NST1-CM-W	0	0	NaN	NaN
SPB1-CM-W	NST2-CM-W	0	0	NaN	NaN
SPB1-CM-W	STN-CM-C	0	0	NaN	NaN
SPB1-CM-W	KSN2-CG-C	−0.26	0	−Inf	NaN
SPB1-CM-W	LAO-CM-C	−0.033	0	−Inf	NaN
SPB2-CM-W	NPT1-CM-W	0	0	NaN	NaN
SPB2-CM-W	NPT2-CM-W	0	0	NaN	NaN
SPB2-CM-W	NST1-CM-W	0	0	NaN	NaN
SPB2-CM-W	NST2-CM-W	0	0	NaN	NaN
SPB2-CM-W	STN-CM-C	0	0	NaN	NaN
SPB2-CM-W	KSN2-CG-C	−0.26	0	−Inf	NaN
SPB2-CM-W	LAO-CM-C	−0.33	0	−Inf	NaN
NPT1-CM-W	NPT2-CM-W	0	0	NaN	NaN
NPT1-CM-W	NST1-CM-W	0	0	NaN	NaN
NPT1-CM-W	NST2-CM-W	0	0	NaN	NaN
NPT1-CM-W	STN-CM-C	0	0	NaN	NaN
NPT1-CM-W	KSN2-CG-C	−0.26	0	−Inf	NaN
NPT1-CM-W	LAO-CM-C	−0.033	0	−Inf	NaN
NPT2-CM-W	NST1-CM-W	0	0	NaN	NaN
NPT2-CM-W	NST2-CM-W	0	0	NaN	NaN
NPT1-CM-W	STN-CM-C	0	0	NaN	NaN
NPT2-CM-W	KSN2-CG-C	−0.26	0	−Inf	NaN
NPT2-CM-W	LAO-CM-C	−0.033	0	−Inf	NaN

Population 1	Population 2	Difference of mean	Standard error	*t*-test	*p*-value
NST1-CM-W	NST2-CM-W	0	0	NaN	NaN
NST1-CM-W	STN-CM-C	0	0	NaN	NaN
NST1-CM-W	KSN2-CG-C	−0.26	0	−Inf	NaN
NST1-CM-W	LAO-CM-C	−0.033	0	−Inf	NaN
NST2-CM-W	STN-CM-C	0	0	NaN	NaN
NST2-CM-W	KSN2-CG-C	−0.26	0	−Inf	NaN
NST2-CM-W	LAO-CM-C	−0.033	0	−Inf	NaN
STN-CM-C	KSN2-CG-C	−0.26	0	−Inf	NaN
STN-CM-C	LAO-CM-C	−0.033	0	−Inf	NaN
KSN2-CG-C	LAO-CM-C	0.227	0	−Inf	NaN

Inf, infinitive; NaN, not a number

Table S5 Comparison of observed heterozygosity (H_o) for the *GH1* gene between catfish populations

Population 1	Population 2	Difference of mean	Standard Error	*t*-test	*p*-value
SNK3-CM-W	SBR-CM-C	0.012	0	Inf	NaN
SNK3-CM-W	KSN1-CG-C	−0.261	0	−Inf	NaN
SNK3CM-W	NYK-CG-C	−0.117	0	−Inf	NaN
SNK3-CM-W	SNK4-CM-C	0.012	0	Inf	NaN
SNK3-CM-W	SNK2-CM-W	0.012	0	Inf	NaN
SNK3-CM-W	SNK1-CM-W	0.012	0	Inf	NaN
SNK3-CM-W	SPB1-CM-W	0.012	0	Inf	NaN
SNK3-CM-W	SPB2-CM-W	0.012	0	Inf	NaN
SNK3-CM-W	NPT1-CM-W	0.012	0	Inf	NaN
SNK3-CM-W	NPT2-CM-W	0.012	0	Inf	NaN
SNK3-CM-W	NST1-CM-W	0.012	0	Inf	NaN
SNK3-CM-W	NST2-CM-W	0.012	0	Inf	NaN
SNK3-CM-W	STN-CM-C	0.012	0	Inf	NaN
SNK3-CM-W	KSN2-CG-C	−0.236	0	−Inf	NaN
SNK3-CM-W	LAO-CM-C	−0.021	0	−Inf	NaN
SBR-CM-C	KSN1-CG-C	−0.273	0	−Inf	NaN
SBR-CM-C	NYK-CG-C	−0.129	0	−Inf	NaN
SBR-CM-C	SNK4-CM-C	0	0	NaN	NaN
SBR-CM-C	SNK2-CM-W	0	0	NaN	NaN
SBR-CM-C	SNK1-CM-W	0	0	NaN	NaN
SBR-CM-C	SPB1-CM-W	0	0	NaN	NaN
SBR-CM-C	SPB2-CM-W	0	0	NaN	NaN
SBR-CM-C	NPT1-CM-W	0	0	NaN	NaN
SBR-CM-C	NPT2-CM-W	0	0	NaN	NaN
SBR-CM-C	NST1-CM-W	0	0	NaN	NaN
SBR-CM-C	NST2-CM-W	0	0	NaN	NaN
SBR-CM-C	STN-CM-C	0	0	NaN	NaN
SBR-CM-C	KSN2-CG-C	−0.248	0	−Inf	NaN
SBR-CM-C	LAO-CM-C	−0.033	0	−Inf	NaN
KSN1-CG-C	NYK-CG-C	0.144	0	Inf	NaN
KSN1-CG-C	SNK4-CM-C	0.273	0	Inf	NaN
KSN1-CG-C	SNK2-CM-W	0.273	0	Inf	NaN
KSN1-CG-C	SNK1-CM-W	0.273	0	Inf	NaN
KSN1-CG-C	SPB1-CM-W	0.273	0	Inf	NaN
KSN1-CG-C	SPB2-CM-W	0.273	0	Inf	NaN
KSN1-CG-C	NPT1-CM-W	0.273	0	Inf	NaN

Population 1	Population 2	Difference of mean	Standard Error	*t*-test	*p*-value
KSN1-CG-C	NPT2-CM-W	0.273	0	Inf	NaN
KSN1-CG-C	NST1-CM-W	0.273	0	Inf	NaN
KSN1-CG-C	NST2-CM-W	0.273	0	Inf	NaN
KSN1-CG-C	STN-CM-C	0.273	0	Inf	NaN
KSN1-CG-C	KSN2-CG-C	0.025	0	Inf	NaN
KSN1-CG-C	LAO-CM-C	0.24	0	Inf	NaN
NYK-CG-C	SNK4-CM-C	0.129	0	Inf	NaN
NYK-CG-C	SNK2-CM-W	0.129	0	Inf	NaN
NYK-CG-C	SNK1-CM-W	0.129	0	Inf	NaN
NYK-CG-C	SPB1-CM-W	0.129	0	Inf	NaN
NYK-CG-C	SPB2-CM-W	0.129	0	Inf	NaN
NYK-CG-C	NPT1-CM-W	0.129	0	Inf	NaN
NYK-CG-C	NPT2-CM-W	0.129	0	Inf	NaN
NYK-CG-C	NST1-CM-W	0.129	0	Inf	NaN
NYK-CG-C	NST2-CM-W	0.129	0	Inf	NaN
NYK-CG-C	STN-CM-C	0.129	0	Inf	NaN
NYK-CG-C	KSN2-CG-C	−0.119	0	−Inf	NaN
NYK-CG-C	LAO-CM-C	0.09	0	Inf	NaN
SNK4-CM-C	SNK2-CM-W	0	0	NaN	NaN
SNK4-CM-C	SNK1-CM-W	0	0	NaN	NaN
SNK4-CM-C	SPB1-CM-W	0	0	NaN	NaN
SNK4-CM-C	SPB2-CM-W	0	0	NaN	NaN
SNK4-CM-C	NPT1-CM-W	0	0	NaN	NaN
SNK4-CM-C	NPT2-CM-W	0	0	NaN	NaN
SNK4-CM-C	NST1-CM-W	0	0	NaN	NaN
SNK4-CM-C	NST2-CM-W	0	0	NaN	NaN
SNK4-CM-C	STN-CM-C	0	0	NaN	NaN
SNK4-CM-C	KSN2-CG-C	−0.248	0	−Inf	NaN
SNK4-CM-C	LAO-CM-C	−0.033	0	−Inf	NaN
SNK2-CM-W	SNK1-CM-W	0	0	NaN	NaN
SNK2-CM-W	SPB1-CM-W	0	0	NaN	NaN
SNK2-CM-W	SPB2-CM-W	0	0	NaN	NaN
SNK2-CM-W	NPT1-CM-W	0	0	NaN	NaN
SNK2-CM-W	NPT2-CM-W	0	0	NaN	NaN
SNK2-CM-W	NST1-CM-W	0	0	NaN	NaN
SNK2-CM-W	NST2-CM-W	0	0	NaN	NaN
SNK2-CM-W	STN-CM-C	0	0	NaN	NaN

Population 1	Population 2	Difference of mean	Standard Error	*t*-test	*p*-value
SNK2-CM-W	KSN2-CG-C	−0.248	0	−Inf	NaN
SNK2-CM-W	LAO-CM-C	−0.033	0	−Inf	NaN
SNK1-CM-W	SPB1-CM-W	0	0	NaN	NaN
SNK1-CM-W	SPB2-CM-W	0	0	NaN	NaN
SNK1-CM-W	NPT1-CM-W	0	0	NaN	NaN
SNK1-CM-W	NPT2-CM-W	0	0	NaN	NaN
SNK1-CM-W	NST1-CM-W	0	0	NaN	NaN
SNK1-CM-W	NST2-CM-W	0	0	NaN	NaN
SNK1-CM-W	STN-CM-C	0	0	NaN	NaN
SNK1-CM-W	KSN2-CG-C	−0.248	0	−Inf	NaN
SNK1-CM-W	LAO-CM-C	−0.033	0	−Inf	NaN
SPB1-CM-W	SPB2-CM-W	0	0	NaN	NaN
SPB1-CM-W	NPT1-CM-W	0	0	NaN	NaN
SPB1-CM-W	NPT2-CM-W	0	0	NaN	NaN
SPB1-CM-W	NST1-CM-W	0	0	NaN	NaN
SPB1-CM-W	NST2-CM-W	0	0	NaN	NaN
SPB1-CM-W	STN-CM-C	0	0	NaN	NaN
SPB1-CM-W	KSN2-CG-C	−0.248	0	−Inf	NaN
SPB1-CM-W	LAO-CM-C	−0.033	0	−Inf	NaN
SPB2-CM-W	NPT1-CM-W	0	0	NaN	NaN
SPB2-CM-W	NPT2-CM-W	0	0	NaN	NaN
SPB2-CM-W	NST1-CM-W	0	0	NaN	NaN
SPB2-CM-W	NST2-CM-W	0	0	NaN	NaN
SPB2-CM-W	STN-CM-C	0	0	NaN	NaN
SPB2-CM-W	KSN2-CG-C	−0.248	0	−Inf	NaN
SPB2-CM-W	LAO-CM-C	−0.033	0	−Inf	NaN
NPT1-CM-W	NPT2-CM-W	0	0	NaN	NaN
NPT1-CM-W	NST1-CM-W	0	0	NaN	NaN
NPT1-CM-W	NST2-CM-W	0	0	NaN	NaN
NPT1-CM-W	STN-CM-C	0	0	NaN	NaN
NPT1-CM-W	KSN2-CG-C	−0.248	0	−Inf	NaN
NPT1-CM-W	LAO-CM-C	−0.033	0	−Inf	NaN
NPT2-CM-W	NST1-CM-W	0	0	NaN	NaN
NPT2-CM-W	NST2-CM-W	0	0	NaN	NaN
NPT1-CM-W	STN-CM-C	0	0	NaN	NaN
NPT2-CM-W	KSN2-CG-C	−0.248	0	−Inf	NaN
NPT2-CM-W	LAO-CM-C	−0.033	0	−Inf	NaN

**Population 1**	**Population 2**	**Difference of mean**	**Standard Error**	***t*-test**	***p*-value**
NST1-CM-W	NST2-CM-W	0	0	NaN	NaN
NST1-CM-W	STN-CM-C	0	0	NaN	NaN
NST1-CM-W	KSN2-CG-C	−0.248	0	−Inf	NaN
NST1-CM-W	LAO-CM-C	−0.033	0	−Inf	NaN
NST2-CM-W	STN-CM-C	0	0	NaN	NaN
NST2-CM-W	KSN2-CG-C	−0.248	0	−Inf	NaN
NST2-CM-W	LAO-CM-C	−0.033	0	−Inf	NaN
STN-CM-C	KSN2-CG-C	−0.248	0	−Inf	NaN
STN-CM-C	LAO-CM-C	−0.033	0	−Inf	NaN
KSN2-CG-C	LAO-CM-C	0.215	0	Inf	NaN

Inf, infinitive; not a number

Table S6 Genetic differentiation between catfish populations in Thailand and Laos based on the *GH1* sequences

Population 1	Population 2	F_{ST}	F_{ST}^{ENA}
SBR-CM-C	SNK3-CM-W	−0.012	−0.013
KSN1-CG-C	SBR-CM-C	0.717	0.697
NYK-CG-C	KSN1-CG-C	0.025	0.024
SNK4-CM-C	NYK-CG-C	0.899	0.873
SNK2-CM-W	SNK4-CM-C	−0.009	0.061
SNK1-CM-W	SNK2-CM-W	−0.016	−0.008
SPB1-CM-W	SNK1-CM-W	−0.110	−0.002
SPB1-CM-W	SPB1-CM-W	-	−0.179
NPT1-CM-W	SPB2-CM-W	-	−0.352
NPT2-CM-W	NPT1-CM-W	-	−0.500
NST1-CM-W	NPT2-CM-W	-	−0.352
NST2-CM-W	NST1-CM-W	-	−0.133
STN-CM-C	NST2-CM-W	-	−0.037
KSN2-CG-C	STN-CM-C	0.796	0.790
LAO-CM-C	KSN2-CG-C	0.078	0.082

F_{ST}, pairwise genetic differentiation; F_{ST}^{ENA}, pairwise F_{ST}^{ENA} values with ENA correction for null alleles

Table S7 The results of Analysis of Molecular Variance (AMOVA) for 16 catfish populations in Thailand and Laos

**Source**	**df**	**SS**	**Var**	**% of variance**
Among Population	15	253.104	0.246	78%
Among Individual	573	50.200	0.017	5%
Within Individual	589	32.000	0.054	17%
Total	1177	335.214	0.317	100%

df, degree of freedom*f*);SS, sum of squares); Var, variance components)
